# Does the endometrial thickness on the day of the trigger affect the pregnancy outcomes after fresh cleaved embryo transfer in the clomiphene citrate‐based minimal stimulation cycle?

**DOI:** 10.1002/rmb2.12315

**Published:** 2020-01-06

**Authors:** Seiko Nishihara, Junichiro Fukuda, Kenji Ezoe, Masako Endo, Yuko Nakagawa, Rie Yamadera, Tamotsu Kobayashi, Keiichi Kato

**Affiliations:** ^1^ Kato Ladies Clinic Shinjuku‐ku Tokyo Japan

**Keywords:** clomiphene citrate, embryo transfer, endometrial thickness, ongoing pregnancy, proliferative phase

## Abstract

**Purpose:**

Thin endometrium is often observed after clomiphene citrate (CC) administration for follicular development and is one of the reasons for embryo transfer (ET) cancelation or implantation failure. We retrospectively analyzed whether the endometrial thickness (EMT) on the days of the maturation trigger and ET are predictive factors of pregnancy outcomes after fresh cleaved ET in a CC‐based minimal stimulation cycle (CC‐cycle).

**Methods:**

A total of 746 CC‐cycles in vitro fertilization (IVF), followed by fresh cleaved ET, from November 2018 to March 2019 were analyzed. Associations between the pregnancy outcomes and EMT on the days of the trigger and ET were statistically evaluated.

**Results:**

Although the EMT on the day of ET was not significantly associated with the ongoing pregnancy rate (adjusted odds ratio [AOR], 1.043; *P* = .3251), a decreased EMT on the day of the trigger was significantly associated with a low ongoing pregnancy rate (AOR, 1.154; *P* = .0042). Furthermore, the clinical pregnancy rate was significantly lower when the EMT was <7 mm on the day of the trigger during the CC‐cycle.

**Conclusions:**

These results suggest that measurement of the EMT on the day of the trigger could be effective for predicting the pregnancy outcomes after fresh cleaved ET during the CC‐cycle.

## 1 INTRODUCTION

In the human endometrium, steroid hormones secreted from the ovaries cause repeated cell division, differentiation, and degeneration. The menstrual cycle is divided into a menstrual phase, a proliferation phase, which occurs before ovulation, and a secretory phase, which occurs after ovulation. In the secretory phase, the functional layer of the endometrium is thickened by the actions of estrogen and progesterone to prepare for embryo implantation. However, if embryo implantation does not occur, the functional layer of the endometrium detaches, and menstruation begins. In the menstrual and proliferation phase, the functional layer is repaired under the effect of estrogen, and once again the endometrial thickness (EMT) increases to prepare for embryo implantation.^1^, ^2^ Previous reports on assisted reproduction suggest a relationship between pregnancy outcomes and EMT on the day of human chorionic gonadotropin (hCG) administration and embryo transfer (ET) in controlled ovarian stimulation (COS) cycles. A decrease in EMT is associated with reduced likelihood of pregnancy.^3^, ^4^, ^5^, ^6^, ^7^, ^8^, ^9^, ^10^, ^11^, ^12^, ^13^, ^14^ Therefore, in most institutions, EMT on the day of hCG administration and/or ET is often used as an effective predictor for pregnancy outcomes in COS cycles. If the endometrium is thinner than the institutional criteria on the day of ET, the ET is often canceled, and the embryo is frozen for transfer in the next cycle.

Clomiphene citrate (CC) is a drug used in assisted reproduction, mainly minimal stimulation cycle in vitro fertilization.^15^, ^16^, ^17^, ^18^ CC binds to estrogen receptors in the subthalamic area and inhibits the negative feedback of estrogen, thereby inducing the secretion of gonadotropin‐releasing hormones (GnRH) which leads to the increase of follicle‐stimulating hormone (FSH) and luteinizing hormone (LH). Recently, it was reported that the use of CC in the minimal stimulation cycle results in similar fertilization rates, embryonic developmental rates, and pregnancy rates as that reported for the controlled ovarian stimulation cycle.^19^ Moreover, the reduced rate of ovarian hyperstimulation syndrome and decreased hormone administration decrease the burden to the patients in the minimal stimulation cycle.^18^ However, the EMT is markedly decreased in CC‐based minimal stimulation cycles. Thus, in some patients, ET is canceled on the scheduled day because of endometrium thinning to avoid the risk of implantation failure.^20^, ^21^, ^22^, ^23^ As such cancelations and implantation failures are burdens to the patient, both physically and economically, these adverse effects of CC on the endometrium should be predicted early and avoided. Therefore, in the present study, we retrospectively analyzed the relationships between EMT on the day of the maturation trigger and pregnancy outcomes after fresh cleaved ET.

## 2 MATERIALS AND METHODS

### 2.1 Study patients

A total of 792 treatment cycles in 792 women who underwent their first oocyte retrieval during a CC‐based minimal stimulation cycle followed by fresh cleaved ET on day 2 at the Kato Ladies Clinic were performed between November 2018 and March 2019 were conducted. Cycles in which an embryo derived from an immature oocyte was transferred were excluded (n = 46). Finally, the clinical records of 746 cycles were retrospectively reviewed.

### 2.2 Minimal ovarian stimulation cycle in vitro fertilization

The detailed protocol for minimal stimulation with CC has been previously reported.^9^, ^24^, ^25^, ^26^ In brief, CC (50‐100 mg/d; Fuji Pharma Co., Ltd.) was orally administered, with an extended regimen, from day 3 of the retrieval cycle to the day before induction of final oocyte maturation. The EMT was measured by ultrasound on the day of the trigger. Ovulation triggering was performed using a nasal spray containing the gonadotropin‐releasing hormone agonist, buserelin (Suprecur; Mochida Pharmaceutical Co., Ltd. or Buserecur; Fuji Pharma Co., Ltd.).

Oocyte retrieval was usually performed 30‐36 hours after triggering using a 21‐G needle (Kitazato Corporation), generally without anesthesia or follicular flushing. Cumulus‐oocyte complexes (COCs) were collected, washed, and then transferred to human tubal fluid (HTF) medium (Kitazato Corporation) with paraffin oil at 5% CO_2_ in air at 37°C for culturing, until either conventional in vitro fertilization (cIVF) was performed 3 hours later or, in cases of intracytoplasmic sperm injection (ICSI), denudation was performed 4 hours after oocyte retrieval.^25^ For ICSI, cumulus cells surrounding the oocytes were removed, and the denuded oocytes were cultured in HTF medium covered by paraffin oil for 1 hour before ICSI.

Sperm samples were collected by masturbation and washed by centrifugation through 70% and 90% density gradients (Isolate; Irvine Scientific). Prepared sperm was cultured in HTF medium at 5% CO_2_ in air at 37°C until use.

### 2.3 Conventional insemination, intracytoplasmic sperm injection, and embryo culture

For cases of cIVF, HTF medium supplemented with 10% serum substitute (Irvine Scientific) was used as a fertilization medium.^13^, ^14^ COCs were cultured with sperm (100 000 sperm/mL) at 5% CO_2_ in air at 37°C. Extrusion of the second polar body was confirmed at 5 hours after insemination (day 0), following the removal of cumulus cells. The oocytes were individually cultured in an EmbryoSlide (Vitrolife, Inc) in 180‐µL medium drops (ONESTEP medium; Nakamedical, Inc) with paraffin oil. In cases of ICSI, oocytes were immediately placed in an EmbryoSlide after sperm injection. All embryos were cultured at 37°C (gas phase: 5% O_2_, 6% CO_2_, and 89% N_2_) in an Embryoscope + time‐lapse incubator (Vitrolife). Fertilization was assessed by visualization of two pronuclei (PN) with the use of EmbryoViewer software (Vitrolife).

### 2.4 Embryo transfer

Single fresh cleaved ETs were performed on day 2 after oocyte retrieval as previously described.^13^, ^24^ The EMT was measured by ultrasound immediately before the ET procedure. Dydrogesterone (30 mg/d; Mylan EPD GK) was routinely orally administered during the early luteal phase after the ET. The clinical pregnancy rate and ongoing pregnancy rate were defined according to the ultrasonographic observation of a gestational sac at 3 weeks after ET and the observation of a fetal heartbeat at 5 weeks after ET, respectively.

### 2.5 Statistical analyses

All statistical analyses were performed using JMP software (SAS). Proportion data were analyzed using the Cochran‐Armitage test for trends and Pearson's chi‐squared tests. Continuous parameters were compared using the Student's *t* test or one‐way analysis of variance (ANOVA), with significance evaluated using Tukey's test for post hoc analysis. Logistic regression was used to assess the contributing strength of the parameters associated with pregnancy outcomes. Odds ratios and adjusted odds ratios (AORs) are reported with 95% confidence intervals (CIs) for each group. A receiver operating characteristic (ROC) analysis was also performed, and the area under the ROC curve (AUC) was calculated. A *P* value of <.05 was considered statistically significant.

## 3 RESULTS

Demographic patient and cycle characteristics are shown in Table 1. During the study period, 746 patients underwent 746 oocyte retrieval cycles followed by the transfer of a single fresh embryo derived from a mature oocyte. The mean female and male ages were 38.1 ± 0.1 and 40.2 ± 0.2, respectively. The EMT on the days of the trigger and ET were 6.9 ± 0.1 mm (5‐14 mm) and 10.2 ± 0.2 mm (8‐20 mm), respectively. Among the 746 embryos transferred, 301 (40.4%) were produced by cIVF and 445 (59.6%) were produced by ICSI. The rates of clinical pregnancy and ongoing pregnancy after transfer were 23.3% (174/746) and 20.5% (153/746), respectively.

**Table 1:** Patient characteristics and pregnancy outcomes

No. of patients, n	746
Female age, mean ± SEM (range)	38.1 ± 0.1 (24‐48)
Male age, mean ± SEM (range)	40.2 ± 0.2 (26‐66)
No. of previous ET cycles, mean ± SEM (range)	0.4 ± 0.0 (0‐3)
Infertility cause, n (%)	
Ovulation factor	22 (3.0)
Oviduct factor	5 (0.7)
Endometrial factor	91 (12.2)
Male factor	138 (18.5)
Combination	32 (4.3)
Unexplained	458 (61.4)
No. of ET cycles	746
Endometrial thickness on the day of the trigger (mm)	6.9 ± 0.1 (5‐14)
Endometrial thickness on the day of ET (mm)	10.2 ± 0.2 (8‐20)
Insemination	
cIVF	301 (40.4)
ICSI	445 (59.6)
Morphological grade of the transferred embryos, n (%)	
Grade 1	107 (14.3)
Grade 2	274 (36.7)
Grade 3	359 (48.1)
Grade 4	6 (0.8)
Clinical pregnancy, n (%)	174 (23.3)
Ongoing pregnancy, n (%)	153 (20.5)

Abbreviations: cIVF, conventional in vitro fertilization; ET, embryo transfer; ICSI, intracytoplasmic sperm injection.

Patients were stratified into four groups according to the EMT on the day of the trigger: Group A, EMT < 6 mm; B, 6 mm ≤ EMT < 7 mm; C, 7 mm ≤ EMT < 8 mm; and D, 8 mm ≤ EMT (Table 2). Both clinical and ongoing pregnancy rates after fresh ET significantly increased as the EMT increased from group A to group D. The Cochran‐Armitage test confirmed significant impairments in both clinical and ongoing pregnancy rates with decreasing EMT on the day of the trigger (*P* < .0004 and *P* = .0162, respectively).

**Table 2:** Pregnancy outcomes after fresh cleaved embryo transfer, stratified by the endometrial thickness on the day of the trigger in CC‐cycle

	No. of cycles	Female age	Clinical pregnancy (%)	Ongoing pregnancy (%)
Group A (EMT < 6 mm)	149	38.2 ± 0.3	25 (16.8)^a^	22 (14.8)^a^
Group B (6 mm ≤ EMT < 7 mm)	177	38.0 ± 0.3	35 (19.8)^a,b^	32 (18.1)^a,b^
Group C (7 mm ≤ EMT < 8 mm)	178	38.2 ± 0.3	46 (25.8)^b,c^	41 (23.0)^a,b^
Group D (8 mm ≤ EMT)	242	37.9 ± 0.3	68 (28.1)^c^	58 (24.0)^b^
Cochran‐Armitage trend test (*P* value)	—	—	.0040	.0162

^a‐c^Different superscript letters indicate a significant difference at *P* < .05.

Patients were also stratified into four groups according to the EMT on the day of ET: Group A, 8 mm ≤ EMT < 9 mm; B, 9 mm ≤ EMT < 10 mm; C, 10 mm ≤ EMT < 11 mm; and D, 11 mm ≤ EMT (Table 3). The clinical pregnancy rate was significantly associated with the EMT on the day of ET (*P* = .0260). Furthermore, the ongoing pregnancy rate tended to decrease as the EMT on the day of ET decreased (*P* = .0752).

**Table 3:** Pregnancy outcomes after fresh cleaved embryo transfer, stratified by the endometrial thickness on the day of the trigger in CC‐cycle

	No. of cycles	Female age	Clinical pregnancy (%)	Ongoing pregnancy (%)
Group A (8 mm ≤ EMT < 9 mm)	119	39.2 ± 0.3^a^	20 (16.8)^a^	19 (16.0)^a^
Group B (9 mm ≤ EMT < 10 mm)	145	37.9 ± 0.3^b^	26 (17.9)^a^	22 (15.2)^a^
Group C (10 mm ≤ EMT < 11 mm)	272	38.2 ± 0.2^b^	69 (25.4)^a,b^	57 (21.0)^a,b^
Group D (11 mm ≤ EMT)	210	37.3 ± 0.3^b^	59 (28.1)^b^	55 (26.2)^b^
Cochran‐Armitage trend test (*P* value)	—	—	.0260	.0752

^a‐b^Different superscript letters indicate a significant difference at *P* < .05.

The univariate logistic analysis revealed significant associations between the ongoing pregnancy rate and female age, male age, and the EMT on the days of the trigger and ET (Table 4). To adjust for potential statistical co‐founding biases, a multivariate logistic regression analysis was performed. Decreased EMT on the day of the trigger was significantly associated with a lower ongoing pregnancy rate (AOR, 1.154; 95% CI, 1.046‐1.274; *P* = .0042), even after adjustment for confounders. However, no statistical association was observed between the ongoing pregnancy rate and the EMT on the day of ET after adjustment for confounders (AOR, 1.043; 95% CI, 0.958‐1.136; *P* = .3251).

**Table 4:** Multivariate logistic regression analysis for ongoing pregnancy

	Univariate analysis				Multivariate analysis			
	Odds ratio	95% CI	*P* value	AUC	Adjusted odds ratio	95% CI	*P* value	AUC
Female age	0.877	0.842‐0.913	<.0001	0.657	0.904	0.857‐0.951	.0001	0.675
Male age	0.931	0.902‐0.960	<.0001	0.615	0.981	0.946‐1.017	.3087	
No. of previous ET	0.954	0.739‐1.212	.7093	0.502	1.034	0.791‐1.332	.7958	
Insemination								
cIVF	Reference	—		0.523	Reference	—		
ICSI	0.820	0.593‐1.138	.2349		0.768	0.549‐1.075	.1240	
Morphological grade								
Grade 1	Reference	—	—	0.522	Reference	—	—	
Grade 2	0.987	0.611‐1.622	.9569		1.171	0.704‐1.945	.5433	
Grade 3	0.847	0.531‐1.376	.4945		0.910	0.554‐1.496	.7111	
Grade 4	0.448	0.023‐2.661	.4197		0.458	0.052‐4.002	.4805	
EMT on the day of the trigger (mm)	1.213	1.116‐1.320	<.0001	0.594	1.154	1.046‐1.274	.0042	
EMT on the day of ET (mm)	1.116	1.031‐1.206	.0057	0.565	1.043	0.958‐1.136	.3251	

Abbreviations: AUC, the area under the ROC curve; CI, confidential intervals; cIVF, conventional in vitro fertilization; EMT, endometrial thickness; ET, embryo transfer; ICSI, intracytoplasmic sperm injection.

**Figure 1:**
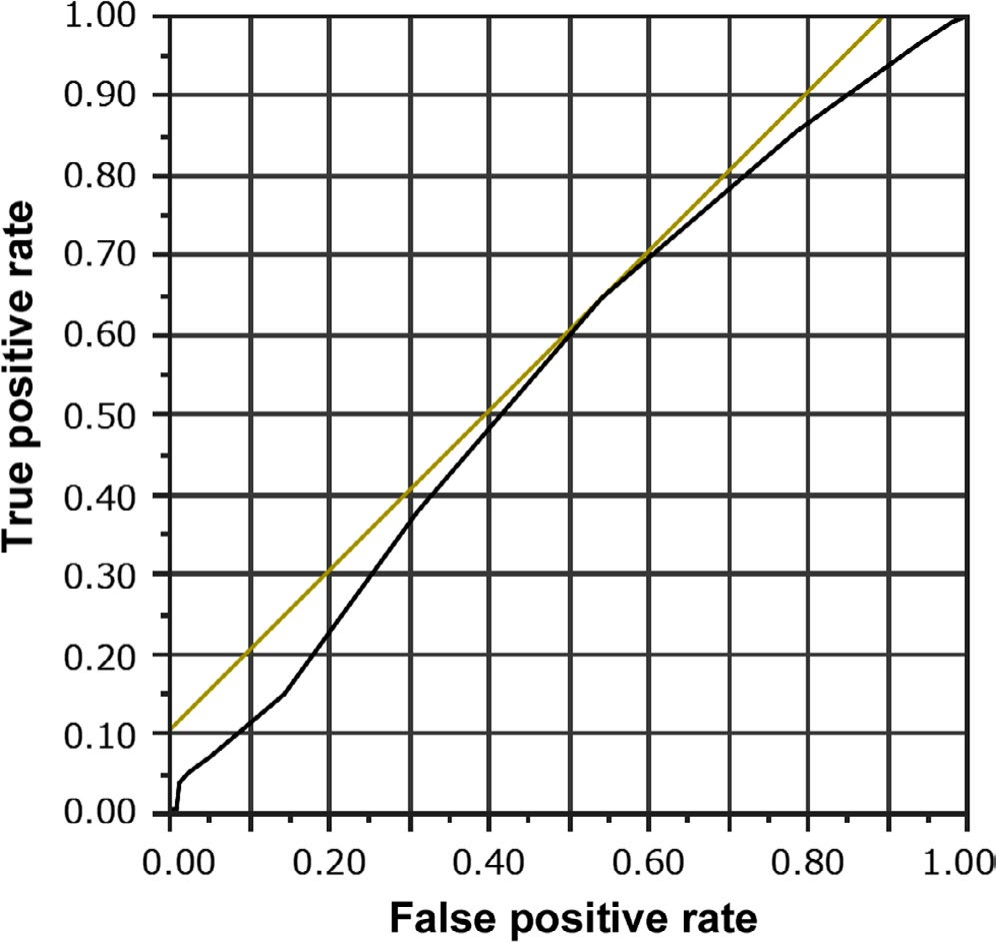
A receiver operating characteristic curve of the endometrial thickness on the day of the trigger for the ongoing pregnancy rate after single fresh cleaved embryo transfers

Furthermore, ROC analysis revealed that for a successful ongoing pregnancy, the optimal cut‐off value of the EMT on the day of the trigger was 7 mm (AUC, 0.594; Figure 1, Table 4). The clinical and ongoing pregnancy rates were significantly lower when the EMT on the day of the trigger was <7 mm than when the EMT was >7 mm (Table 5).

**Table 5:** Pregnancy outcomes after fresh cleaved embryo transfer, stratified by the cut‐off value of endometrial thickness on the day of the trigger

	EMT on the day of the trigger <7 mm	EMT on the day of the trigger ≥7 mm
No. of cycles	326	420
Female age	38.1 ± 0.2	38.0 ± 0.2
Clinical pregnancy (%)	60 (18.4)^a^	114 (27.1)^b^
Ongoing pregnancy (%)	54 (16.6)^a^	99 (23.6)^b^

^a‐b^Different superscript letters indicate a significant difference at *P* < .05.

Abbreviation: EMT, endometrial thickness.

## 4 DISCUSSION

Endometrial thinning is often observed during the implantation period after CC administration for follicular development, resulting in a reduced pregnancy rate. Therefore, the question of how to improve the pregnancy rate after fresh cleaved ET in a CC‐based minimal stimulation cycle remains a significant concern in the field of IVF.^9^, ^10^, ^11^, ^12^, ^15^, ^27^ To date, the relationships between the EMT during the proliferation phase in a CC‐based minimal stimulation cycle and ET outcomes have not been clarified. The present study is the first to report a significant association between the EMT on the day of the trigger and pregnancy outcomes after ET in a CC‐based minimal stimulation cycle.

Previous studies reported that pregnancy rates after ET are reduced when the EMT on the day of transfer (ie, in the secretary phase) is ≤7−8 mm.^4^, ^7^ In the present study, ETs were performed in patients with an EMT ≥8 mm on the day of ET, as based on previous reports, it is highly likely that the endometrium would be receptive to embryo implantation. However, the present study results demonstrate that the EMT on the day of trigger is more tightly associated with pregnancy outcomes than is the EMT on the day of ET in CC‐based minimal stimulation cycles. Normally, the functional layer of the endometrium is quickly repaired after menstruation. During this endometrial repair period, cell proliferation is promoted by estrogen, mediated via estrogen receptor α (ERα), and reportedly peaks at around 8‐10 days from the onset of mensuration.^1^, ^28^, ^29^ In addition, it has been shown that exposure to estrogen for at least 5 days is required for endometrium thickening and the establishment of implantation.^30^, ^31^ In the present study, the estrogen antagonist, CC, was administered on consecutive days, from the menstrual phase to the day of the trigger. Thus, ERα signaling in the endometrial tissue might have been impaired during the proliferation phase, likely inhibiting endometrial repair in patients with a thin endometrium on the day of the trigger. In addition, it has been reported that exposure to estrogen during the proliferation phase increases the sensitivity to progesterone and thickening of the functional layer in the implantation phase.^1^ Based on the results of the present ROC analysis, it is evident that the pregnancy rate after ET is significantly reduced when the EMT on the day of the trigger is thinner than 7 mm. This suggests that sensitivity toward progesterone, as well as embryo reception, is likely decreased in patients in whom the EMT is not repaired to ≥7 mm by the day of the trigger, even if the EMT on the day of ET reaches ≥8 mm.

There are some concerns in the present study. The AUC value of the EMT on the day of the trigger for the ongoing pregnancy after the embryo transfer might not be high enough to predict pregnancy outcomes using only this factor. On the other hand, the establishment of implantation and pregnancy after Day 2 ET is more affected by embryonic factor, endometrial factor, and their interaction, compared with the blastocyst transfer. The embryonic development to the blastocyst stage and the endometrial transition to the receptive phase need to occur simultaneously after the cleaved embryo transfer. Therefore, it is considered that the prediction of the pregnancy outcomes after Day 2 ET would be more difficult than blastocyst transfer, which means that the AUC value might be lower than that in the case of the blastocyst transfer. We calculated the AUC of the female age, male age, number of previous ETs, insemination method, embryonic morphological grade, EMT on the day of the trigger, and EMT on the day of ET for the ongoing pregnancy rate (Table 4). The highest AUC was observed in female age (0.657), and the second highest was in male age (0.615). Although it is well known that a patient's age strongly associates with the IVF outcomes, the AUC value of female and male ages also is not high in the case of Day 2 ET. The AUC of EMT on the day of the trigger was the third highest parameter, and its predictive potential was higher than that of the number of previous ETs, insemination method, embryonic morphological grade, and EMT on the day of ET. Actually, the clinical and ongoing pregnancy rates were significantly lower when the EMT on the day of the trigger was <7 mm than when the EMT was >7 mm (Table 5). Therefore, we consider that the EMT on the day of the trigger can be used as a predictive factor and the combined use of several factors, including the EMT on the day of the trigger, could improve the pregnancy outcomes after Day 2 ET. As mentioned above, however, the AUC value of the EMT on the day of the trigger for the ongoing pregnancy might not be high enough to predict pregnancy outcomes; therefore, further studies are required to validate whether the cut‐off value (7 mm) of the EMT on the day of the trigger was reliable or not. In addition, the present study comprises a single‐center retrospective study, and therefore, the possibility of selection biases cannot be ruled out. As the study outcomes comprised the clinical and ongoing pregnancy rates, the relationship between the EMT on the day of the trigger and the live birth rate requires further analysis. Furthermore, a molecular biology analysis is required to assess the relationship between the EMT on the day of the trigger and uterine receptivity.

In conclusion, the present results suggest that measurement of the EMT on the day of the trigger in a CC‐based minimal stimulation cycle is useful, as the EMT on the day of the trigger is significantly correlated with pregnancy outcomes after fresh cleaved ET. Moreover, a high pregnancy rate can be achieved in patients with an EMT ≥7 mm on the day of the trigger. Therefore, if the EMT does not reach 7 mm by the day of the trigger, fresh ET should be canceled prior to oocyte retrieval, and the treatment plan should be switched to frozen ET; this would improve the pregnancy outcomes, as well as a decrease the patient burden. Moreover, in general infertility treatments, such as intrauterine insemination or timed intercourse, IVF should be proactively recommended in patients who experience thinning of the endometrium due to a CC treatment, even when CC works effectively in terms of follicular development, as this will produce a shorter route to pregnancy.

## CONFLICT OF INTEREST

The authors have no conflicts of interest to declare.

## HUMAN RIGHTS, INFORMED CONSENT, AND ETHICAL APPROVAL

The study comprised a retrospective cohort study approved by the Institutional Review Board of Kato Ladies Clinic (approval number: 19‐27). Written informed consent for the retrospective analysis of de‐identified data was obtained from all patients undergoing IVF treatment at the center. All procedures followed were in accordance with the Helsinki Declaration of 1964 and its later amendments.
